# Data on the uptake and metabolism of testosterone by the common mussel, *Mytilus* spp.

**DOI:** 10.1016/j.dib.2017.03.040

**Published:** 2017-03-31

**Authors:** Tamar I. Schwarz, Ioanna Katsiadaki, Benjamin H. Maskrey, Alexander P. Scott

**Affiliations:** Centre for Environment, Fisheries and Aquaculture Science, Barrack Road, Weymouth, Dorset DT4 8UB, UK.

**Keywords:** Mollusc, Sulphate, Steroid metabolism, androgen

## Abstract

This article provides data in support of the research article entitled “Rapid uptake, biotransformation, esterification and lack of depuration of testosterone and its metabolites by the common mussel, *Mytilus* spp.” (T.I. Schwarz, I. Katsiadaki, B.H. Maskrey, A.P. Scott, 2017) [1]. The uptake of tritiated testosterone (T) from water by mussels is presented. The two main radioactive peaks formed from T and present in the fatty acid ester fraction of mussel tissues were shown to have the same elution positions on a thin layer chromatography plate as 17β-hydroxy-5α-androstan-3-one (DHT) and 5α-androstan-3β,17β-diol (3β,17β-A5α). Reverse phase high performance liquid chromatography of the non-esterified (80% ethanol) fraction of the mussel tissue extracts also presented radioactive peaks at the elution positions of DHT and 3β,17β-A5α. There was no evidence for sulfated T in this fraction. It was shown that aeration led to significant losses of radiolabeled testosterone from the water column.

**Specifications Table**

**Table:** 

Subject area	*Biology*
More specific subject area	*Endocrinology*
Type of data	*Figures*
How data was acquired	*Scintillation counting, HPLC, TLC,*
Data format	*Analyzed*
Experimental factors	*Studying the rate of uptake of tritiated T ([*^*3*^*H]-T) by live mussels and identifying the metabolites produced*
Experimental features	*Measuring rate of disappearance of [*^*3*^*H]-T from water in a vessel containing mussels, extracting and then separating the metabolites in tissue by liquid and thin layer chromatography.*
Data source location	*Portland Harbour, Dorset*
Data accessibility	*Data presented in this article*

## Value of the data

• The data provide supporting evidence to challenge the assumption that T found in the flesh of mussels is necessarily of endogenous origin.
• Identification of DHT and 3β,17β-A5α, both metabolites of T, in the ester fraction of mussel tissues has implications for the accuracy of T quantification in mussels from laboratory exposures and the field.
• The lack of testosterone sulfate in tissue extracts shows that sulfation does not play a major role in T metabolism in mussels, in contrast to E_2_ metabolism.

## 1 Data

**Fig. 1:**
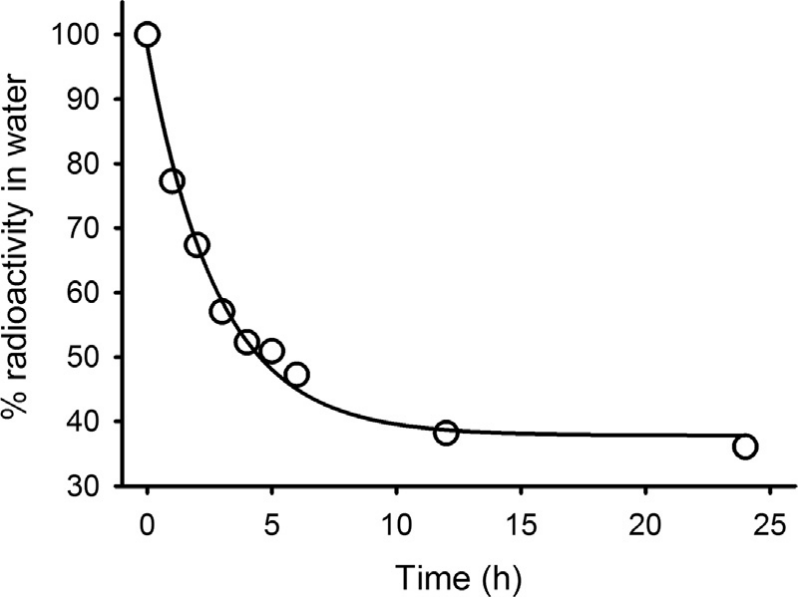
Removal of radioactivity by *Mytilus* spp. during a 24 h exposure (Experiment 3) in a single polythene bag containing 3.6 L water and 18 animals. Data are presented as percentage radiolabel remaining in the water at each sampling point (○). The curve represents the same data fitted to a three parameter exponential decay equation.

**Fig. 2:**
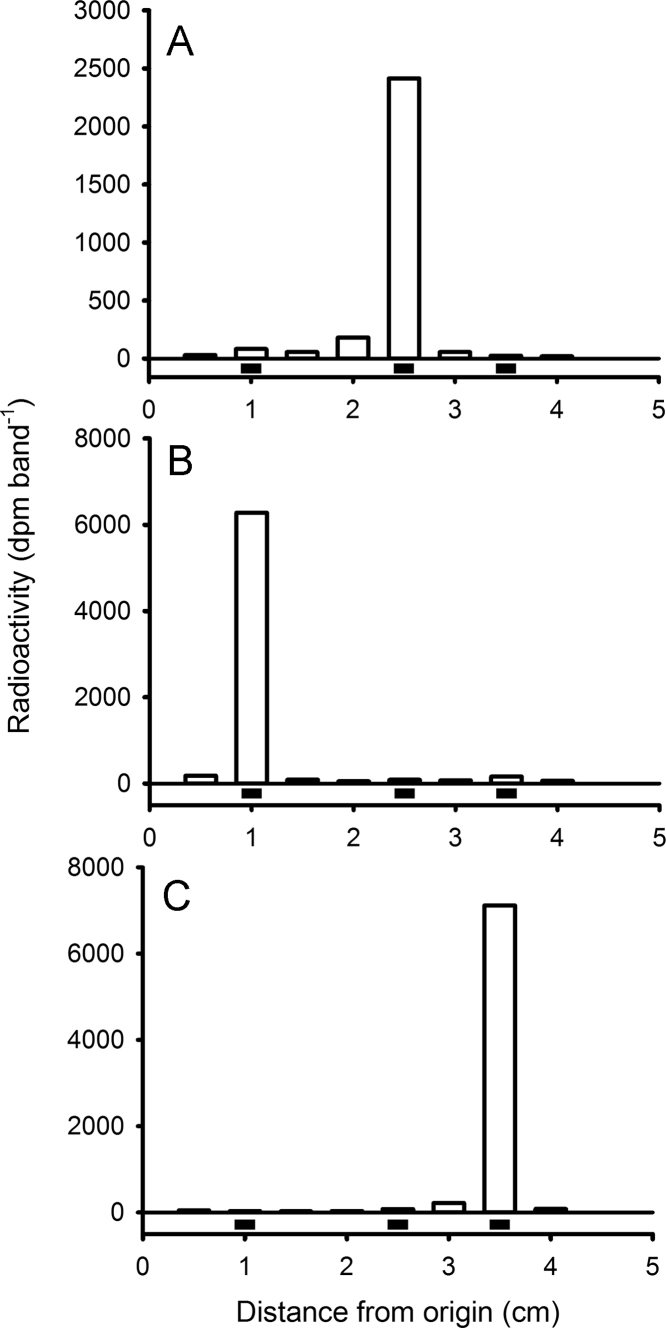
Thin layer chromatography of the radioactive peaks (A: 52 min; B: 54 min and C: 58 min) obtained from the reverse phase HPLC separation of saponified esters (Experiment 1). Standards of 3β,17β-A5α, T and DHT (▄, from left to right) were run concurrently.

**Fig. 3:**
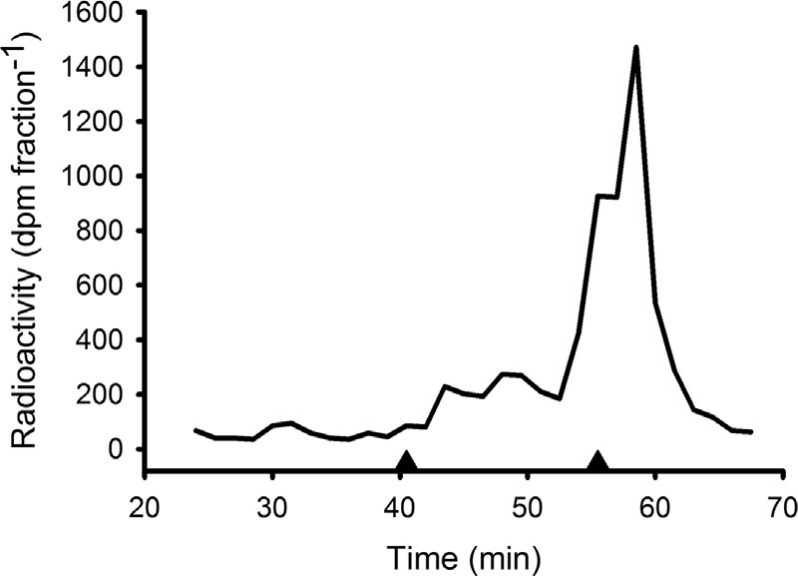
Chromatography on a reverse phase HPLC preparative column of [^3^H]-T radioactivity derived from the 80% ethanol (putative free and sulfate) fraction of a pooled mussel extract (Experiment 3). Also shown are the elution positions of T-S and T standards (▲, from left to right) that were run concurrently and monitored by UV absorption at 245 nm.

**Fig. 4:**
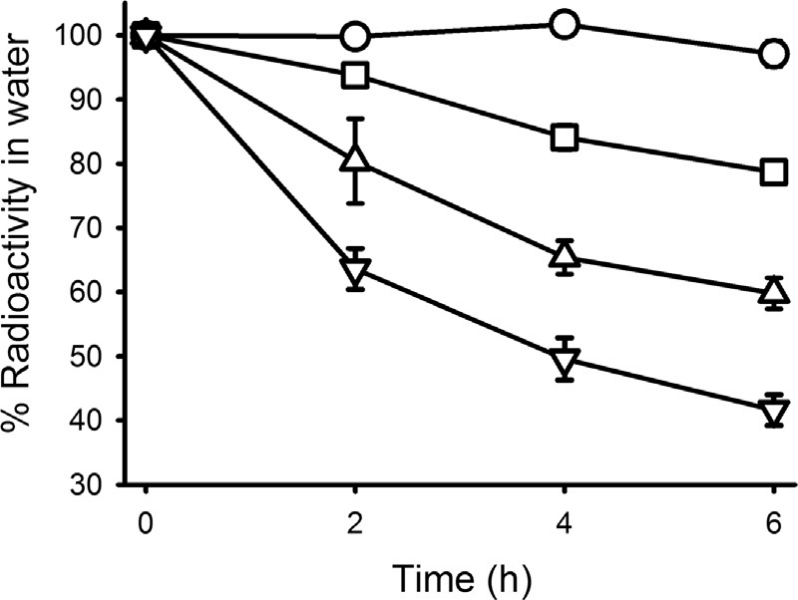
Effect of aeration on the loss of [^3^H]-T radioactivity from seawater. Data are presented as mean percentage±SEM applied radioactivity (n=3) for the following treatments: no aeration (○), low aeration (**□**), medium aeration (△) and high aeration (▽).

The data presented in this article show: the uptake of [^3^H]-T from water by mussels (Fig. 1); thin layer chromatography (TLC) identification of tritiated 3β,17β-A5α, T and DHT in the ester fraction of saponified tissue extracts (Fig. 2); and high performance liquid chromatography (HPLC) identification of [^3^H]-T metabolites in the non-esterified fraction of mussel tissue extracts (Fig. 3). Data indicating the loss of radiolabel from water as a result of aeration are also presented in this article (Fig. 4).

## 2 Experimental design, materials and methods

### 2.1 Thin layer chromatography

The main peaks of radioactivity from the reverse phase HPLC separation of a saponified ester fraction from pooled mussel extract from Experiment 1 [1], were mixed with 5 µg each of T; 3β,17β-A5α and DHT and loaded onto one lane of a TLC plate (catalog no. LK6DF; Whatman Labsales; www.whatman.com; but no longer manufactured). The plate was developed for 45 min with chloroform:methanol (50:2, v-v) as mobile phase. The plate was sprayed with 10% sodium molybdophosphate in ethanol and then heated for 2 min at 150 °C. After marking the positions of the standards, the lanes were divided into 5 mm bands, and the silica gel from each band scraped off the plate. The bands were mixed with 1 mL 80% ethanol and 7 mL scintillation fluid and then scintillation counted for determination of radioactivity.

### 2.2 Effects of aeration on water-borne radiolabel

The experiment consisted of 12 polypropylene vessels containing 300 ml filtered (50 µm) seawater, airstones (mineral plastic) and tubing. In nine of these vessels the tubing was connected via an adjustable valve and an airflow meter to a hobby-grade aquarium air pump. The three non-aerated vessels were gently swirled on an orbital shaker. The valves were adjusted so that three vessels each received low, medium and high rates of airflow (*c*. 40, 240 and 480 mL min^−1^ respectively). After the addition of 3500 dpm mL^−1^ of [^3^H]-T to each vessel, a sample of water (2 mL) was removed (time 0) and aeration was started. Further samples (2 mL) were collected at 2, 4 and 6 h. The water samples were mixed with 10 mL scintillation fluid and counted for 10 min.
